# ‘Never Going to be a Cut Above [the Rest].’ Medical Students’ Perceptions of How Their Personal Protected Characteristics Impacted Surgical Learning

**DOI:** 10.1007/s40670-025-02415-7

**Published:** 2025-05-26

**Authors:** Zara R. Zaccariah, Janet Lefroy, Brianne Wenning

**Affiliations:** 1 Manchester Foundation Trust, Manchester, UK; 2 Keele University, Newcastle-Under-Lyme, UK; 3 King’s College London, London, UK

**Keywords:** Medical Education, Differential Attainment, Surgery, Medical Students, Equality, Discrimination

## Abstract

**Background:**

Differential attainment is a phenomenon studied widely, but the reasons for its existence remain relatively unknown. Discriminatory behaviours have been well documented in the medical field, especially in the surgical environment, and have been shown to impact learning. This study explores experiences of students in the surgical environment which they attribute to their protected characteristics and the impact this had on their learning and attainment.

**Methods:**

A mixed methods modified ethnographic approach involving diary study and semi-structured interviewing was used. Data was analysed using thematic analysis with an interpretive approach. An intersectional lens in analysis provided deeper insight into the individualised experiences of students.

**Results:**

Students believed that others’ reactions to their protected characteristics impacted how they were perceived and treated. They believed this differential treatment impacted their learning and influenced their future career prospects. Participants were not able to adequately compensate for the impact that discriminatory behaviours had on their learning. They were, however, able to suggest areas of improvement which they believed could improve the learning environment.

**Conclusion:**

Our study demonstrated discrepancies in student experience based not only on their individual protected characteristics but also upon the way these intersect with one another. This resulted in suboptimal learning, suggesting that attitudes of surgical placement staff and student peers require review to improve student experience in the learning environment. The findings of this study have implications for the academic performance of students and also their postgraduate performance and patient safety.

## Introduction

Differential attainment is defined as a difference in the average performance of groups with different protected characteristics [1]. In the UK, anti-discrimination law has been subject to legislative and judicial expansion and fragmentation since its origin [2]. For the initial 30 years of this “grounds-based” system, race and sex were the only recognised protected characteristics enshrined in UK law (ibid.). In the years since, seven further characteristics have been included which now comprise the Equality Act 2010. The Equality Act 2010 provides a legal framework which protects individuals against discrimination related to the following characteristics:

- Age
- Disability
- Gender reassignment
- Marriage and civil partnership
- Pregnancy and maternity
- Race
- Religion or belief
- Sex
- Sexual orientation

Studies have shown that students from ethnic minority backgrounds perform worse than their white counterparts in higher education despite controls for prior attainment, socioeconomic status [3], living arrangements, study habits and personality [4]. Research has also shown that female students obtain higher degree classifications than male students [5]. However, the cause of differential attainment remains unclear.

Current evidence points towards the issue in medical education being the learning environment [1]. This highlights the social nature of learning and supports the notion that relationships in the learning environment, such as those between students and their teachers and peers critically affect academic outcomes [6]. This is supported by an international review which found that minority medical students experienced ‘less supportive social and less positive learning environments’ and were more likely to experience discrimination and harassment [7]. This is especially relevant when considering learning in the context of the clinical environment. As Carr and Woodson [8] note, differences in the expectation and treatment of students seem to especially be a problem during the clinical phases of medical school where they become more prominent and noticeable.

The Kennedy report [9] highlighted stark differential attainment in the surgical field in the UK, with lower international medical graduate representation (21.3% in surgery vs 27.5% of all licensed doctors). Only 34.8% of surgical trainees are female in comparison to 56.6% of trainees in other specialities [9]. The surgical workplace has also been described as a high-risk area for bullying, undermining and harassment [10]. Ling et al. [11] found that these behaviours were more likely to affect participants who were trainees and/or female, and consultants were identified as the most common perpetrators. Although this study was conducted in Australia, several UK-based studies report similar findings [12, 13].

Whilst research into differential attainment in medicine [1] and barriers to a surgical career [14] has been conducted, research in medical students is largely limited to the context of race, ethnicity and gender. The impact of other protected characteristics, and the way these might intersect, represents a gap in the literature. This study builds on existing research and addresses this gap by focusing on the experiences of students in the surgical learning environment. To the best of our knowledge, this is the first study of its kind.

The purpose of this research was to gain insight into whether the protected characteristics learners possessed impacted their experiences in the surgical environment. If these experiences were felt by participants to differ from those of others, we explored why this was, how this impacted student learning and future career plans, and whether and how students compensated for any impact. To do so, we used an interpretivist approach to the research question, meaning that we centred the perceptions and interpretations of our participants’ experiences as these describe their socially constructed reality [15]. More specifically, this research ascribes to anthropological ethnographic principles. Ethnography, also referred to as a theory of describing [16] views a phenomenon through the lens of those belonging to a particular social group in a particular cultural setting [17]. This critical focus on the phenomenon in question represents a study *with* people rather than of them, thus including them in the conversation [18]. This ethnographic approach, grounded in an interpretivist paradigm, was crucial for centring the experiences of those who felt they had been ‘othered’ in some form in this learning environment. Through this research, we strive to inform how the surgical environment might be changed to optimise learning for these students in the future.

## Materials and Methods

This study utilised a modified ethnographic approach consisting of both a diary study and semi-structured interviews. By modified, we mean that we employed a short-term ethnography using more intense, innovative research techniques whilst remaining grounded in anthropological principles and an interpretivist paradigm [19]. By doing so, we covered at least 2 weeks of each student’s surgical block as the data collection period and therefore captured the ‘day-to-day’ interactions which they considered relevant in order to get a comprehensive understanding of learners’ experiences.

### Recruitment

Ethical approval was obtained from Keele University’s School-Student Project Ethics Committee (reference 2128) for this Masters project prior to study commencement. The study population was limited to UK medical students who had a surgical rotation during the study period. The study was advertised nationally through institutional correspondence and on social media. Snowball sampling was also utilised, whereby students were asked to ‘refer’ others who fit the criterion for eligibility to participate. The advertisement stated the purpose of the study as “looking at how different characteristics (i.e. gender, ethnicity, age, sexuality) impact how you as students are treated during placements and how this impacts your learning”. Participants were urged to consider that the results of this study might make “a positive difference for future cohorts and […] aid in creating a supportive learning environment which caters to the diversity that is seen in its learners”. Ten students applied to participate and all 10 were recruited.

All participants were encouraged to complete a personal characteristics form (Appendix A) to better facilitate data analysis. In order to preserve student and institutional anonymity, participants were not asked to state their medical school or any other identifying information.

Participants were asked to maintain diaries throughout their surgical rotation and were advised that the diary could be written, audio-recorded or video-recorded. The duration of the diaries would ideally cover a minimum of 2 weeks. Students were provided with a diary guide, which outlined items they should aim to include (Appendix B).

Interviews with participants occurred after they submitted their final diary entry. The interviews followed a guide (Appendix C) to ensure that questions remained relevant and focused. Diary contents were also explored by the interviewer in order to probe deeper into these experiences.

Interviews took place via Microsoft Teams and were video- and audio-recorded. They were then transcribed verbatim and all data was pseudonymised.

Data analysis followed Braun and Clarke’s [20] reflexive thematic analysis. The lead researcher, ZZ, created an open coding framework which was then discussed with JL and BW to ensure concordance and comprehensiveness. The entire research team then engaged in axial and selective coding to categorise codes into higher order themes [21].

Crenshaw’s [22] ‘intersectional sensibility’ was adopted during analysis to ensure that social categories were not considered as being distinct, but rather as dynamic and permeated by other categories. Intersectionality underscores the social construction of social identities, noting how these influence everyday perceptions and experiences and thereby create a social reality [23], thus making it relevant to our overarching framework.

## Results and Discussion

In ethnographic research, results are often discussed as they are presented in order to draw on existing literature to better contextualise data and analytic observations. Each theme will be discussed in turn, using relevant literature and supporting quotes from both the diary entries and semi-structured interviews.

Ten students were recruited. To maintain confidentiality, pseudonyms are used when referring to participants. Table 1 contains information about each participants protected characteristics to provide context to the results.

**Table 1:** Information about study participants

Pseudonym	Protected characteristics
Emma	Aged 23, Female, AFAB*, White British, International student, Christian, Heterosexual, Established middle class†, No disability
Lucy	Aged 24, Female, AFAB*, White British, not an international student, Christian, Heterosexual, Technical middle class†, No disability
Shreya	Aged 27, Female, AFAB*, Sri Lankan British, not an international student, Hindu, Heterosexual, Technical middle class†, No disability
Alysha	Aged 28, Female. AFAB*, Black African, International student, Christian, Heterosexual, Elite†, No disability
Amber	Aged 27, Female, AFAB*, not an international student, other characteristics not disclosed
Luke	Aged 47, Male, AMAB*, White British, not an international student, Atheist, Heterosexual, Technical middle class†, No disability
Sarina	Aged 22, Female, AFAB* British Bangladeshi, not an international student, Muslim, Heterosexual, Technical working class†, No disability
Ken	Aged 28, Male, AMAB*, Black British, not an international student, Christian, Heterosexual, Elite†, No disability
Aisha	Aged 21, Female, AFAB*, British Pakistani, not an international student, Muslim, Heterosexual, New affluent workers†, No disability
Inaya	Aged 22, Female, AFAB*, British Pakistani, not an international student, Muslim, Heterosexual, Technical working class†, No disability

*AFAB (assigned female at birth) and AMAB (assigned male at birth)

^†^Refers to socioeconomic class as defined by the ‘Great British class calculator’ [42] which is based on the works by Savage et al. [43] and as determined by a self-reporting questionnaire

All ten submitted diaries exceeded the 2-week minimum. The maximum diary length was 6 weeks, which was expected as specialty-specific placements typically last 4 to 6 weeks. On average, data from the diaries spanned approximately 3.5 weeks. Nine participants submitted written diaries and one participant chose to submit an audio-recorded diary.

All ten participants were interviewed. Interviews lasted 59 min on average.

By process of inductive thematic analysis, we extracted three super themes from the diaries and interview transcripts—‘why participants felt they were treated differently’, ‘how learning was impacted by differential treatment’ and ‘how did students compensate?’. Each theme comprised of several sub-themes. These results are published in full in a Master’s dissertation “Exploring the impact of ‘personal protected characteristics on student experience during surgical rotations” [24]. Discussion of all three super themes is beyond the scope of this paper so the first theme will not be covered. Instead, the focus will be on how learning was impacted by differential treatment and how students compensated for any impact to learning.

### How Learning Was Impacted by Differential Treatment

#### Approachability, Communication, and Rapport

Participants in this study cited native accents, age, sex, gender and religious views as personal characteristics which they felt were discriminated against. They stated that differential treatment, especially feeling humiliated or bullied for being ‘other’, affected perceptions of placement providers’ approachability, communication and rapport and resulted in poor attendance.

Shreya elaborated on why she avoided attending theatres and stated “…I knew that as soon as I go in there, no one’s gonna help me scrub in. So if you don’t get scrubbed in, you can’t see anything. So…what’s the point standing there if no ones gonna help me.”

Emma also avoided the operating theatre and “…mainly just attended clinics to avoid all of that [bullying] because it’s mainly in the surgeries that this happens.”

Sarina described warning her colleagues to avoid a specific surgeon’s clinic after a discriminatory encounter with her: “When I left, I did tell my peers that I just would not bother going to her clinic if I were you, I’d choose a different one to go to. I have not been back to that one.”

As these quotes highlight, students seemed most concerned about being humiliated in the surgical environment, resulting in the perception that surgeons were less approachable. Previous literature demonstrates how ‘teaching by humiliation’ leads to demoralisation, negatively impacting students’ desires to attend placement [25]. Almost all participants in our study reported witnessing or experiencing such behaviours in the surgical environment. These findings align with decades of literature, in which up to 95% of medical students reported experiencing ‘teaching by humiliation’ behaviours [25, 26].

#### Not Fitting the Stereotype

Some participants felt that their appearance conflicted with that of a stereotypical surgeon. This impacted on how they felt others perceived them.

Aisha, a British Pakistani female believed that “…people view…[surgeons] to be…[a] stereotypical white male who’s well-spoken and I’m…the complete opposite of that if I’m going to be honest, in every aspect.”

Interestingly, Ken (Black British male) felt that he had to put in extra effort because of this: “…I have to go above and beyond and try and be even better than perfect because you know, I almost need to have like a CV and a job application that’s so good they can’t say no whereas someone else can actually be average but still make it.”

Lucy (White British female) echoed the above sentiment: “you could have really good aspirations to go far in your career but then find out actually you’re never going to be [considered] a cut above [the rest]…” This sentiment underscored many participants’ views, giving rise to the title of this manuscript.

These findings are in line with Beagan [27], who found that some of their white participants articulated the privilege of fitting the stereotype of a doctor and describing themselves as “kind of what patients expect to see.” They noted that this allowed them to bond better with colleagues and patients and granted them the automatic status of a doctor, whilst other students had to earn it. This results in underrepresented minority (URM) students attempting to conform to idealistic standards and altering their persona to be more accepted, placing excess pressures on these students. This may result in them not being able to ask for help, which can impede the formation of successful peer support networks that are necessary for optimal academic achievement [28].

#### Confidence and Preparedness

Some participants felt that gender discrimination resulted in decreased opportunities for them, undermining their confidence in their capabilities and perceived preparedness. Impact on confidence was reported primarily by female participants.

Shreya repeatedly voiced concerns over not feeling prepared for her surgical jobs as a junior doctor. She attributed this lack of preparedness and confidence in her skills to the gender discrimination she faced on her surgical placement which resulted in a lack of opportunities to practice, noting it affected “…not only my exam performance, but I think my confidence going into F1/F2 [has been impacted]…I don’t want to be held responsible for disfiguring someone because I didn’t get the time to practice [suturing] in med[ical] school…That’s a really big worry of mine at the moment.”

Amber voiced similar concerns and stated that experiencing discriminatory behaviours would make her question her capabilities and her breadth of knowledge: “Sometimes…I feel like, well, was I good enough? Cause if for me personally, if I went to a theatre and I was treated differently…I would think that it’s because…of all the wrong answers that I’ve given every time they’ve asked me a question. [So] they’re just like, ‘you know what, we’ll, we’ll just leave you out. Surgery is not for you.’”.

Female medical students consistently report more anxiety about their performance, greater worries regarding competency, and less confidence in their abilities in medical school [29]. It has been posited that this is due to medical school experiences; female medical students report higher rates of sexual harassment and gender discrimination, which may raise their anxiety and lower their self-efficacy [29]. This study adds to the scarce existing literature on this topic and supports the notion that the presence of gender disparities in the clinical environment impacts confidence and preparedness and increases anxiety in female medical students.

#### Impact on Career Prospects

A perceived lack of belonging, particularly for those with minority identities, has been found to limit success in academic surgery [30]. Amber described how her disinterest in a surgical career partly stemmed from female underrepresentation:

…if I just had, you know, male role models to look up to…then I’d think well, there’s no one like me in there, how am I gonna fit into all these people. — Amber.

Related justifications were given by Sarina, Alysha and Aisha, who all described how a lack of surgical garments which catered to their Afro-Caribbean hair or faith requirements led to feelings of exclusion and were dissuaders to pursuing surgical careers. This is similar to prior research which has shown that a sense of isolation is correlated with an increased risk of departure from medicine [31]. This sense of isolation is then compounded when these URM professionals ascend through the academic ranks as the underrepresentation magnifies [32]. The physical garments not fitting was perceived as a visual, tangible representation of who ‘fits’ in the surgical world. This infers that this rigid vision of a successful surgeon, i.e. a white male, determines who “belongs” in the surgical environment, excluding and discouraging those who do not fit this image.

#### Impact of Microaggressions

Due to their covert nature, microaggressions can create a degree of uncertainty or paranoia that does not occur with more overt discrimination. This was highlighted by multiple participants. Alysha described the psychological impact of the uncertainty that comes with microaggressions, and reported that this became a distractor during revision:

…when you sit down and study…in the back of your mind, you’re playing that over and over and questioning whether or not you’re overthinking it. So I think it’s more of a distraction than anything else, but it does make it a bit harder to, to perform as well as…you would normally.

This adds to existing literature which finds that experiencing microaggressions can take a toll on the recipient and generate a cognitive load that impacts the students’ ability to perform academically [33]. Furthermore, Alysha’s testimony supports the finding that microaggressions can be pervasive in a learner’s life [34], and are therefore not limited to the context in which they occurred. When students internalise the effects of these microaggressions, they increase stress due to rumination over exposure and feelings of hypervigilance [34].

#### Barriers to Reporting Mean That Discriminatory Treatment Can Continue to Affect Learners

The experiences documented and described by participants impacted their ability to report discriminatory incidents. Students discussed three areas in particular — expressing a lack of faith in institutions, wanting to remain invisible and feeling the effects as a bystander. Each of these will be discussed in more detail below.

- Lack of faith in institutions

A common theme reported by almost all participants was a lack of faith in reporting channels, especially in their respective medical schools. This was often due to a fear of repercussions of reporting senior staff, especially considering that students would still have to interact with them, potentially in examinable circumstances.

I’d rather not get someone annoyed and angry because I don’t know how that’s gonna affect me in the future. [I don’t know] at what point I’m gonna encounter you [again]…And you’re gonna have a say in something that I’m doing, or you’re gonna be able to negatively impact something that I’m doing. — Sarina.

She [female surgeon] basically threatened him [male medical student] and said that you should watch out for your exams and stuff. So then he went and reported it straight away that I don’t want her [to examine me in an] OSCE ever….Since that happened…it’s made me like a bit more wary [that the person I’d report could be a future examiner]. — Inaya.

These findings support existing literature, as students have been found to doubt the anonymity of the reporting process and lack confidence in the certainty that perpetrators would be adequately dealt with [35]. Students also express concern about the potential repercussions on performance evaluations [36]. This suggests that students are underreporting discriminatory incidents, contributing to the lack of intergenerational change. This resilience to cope with perceived ‘minor’ incidents may also contribute to underreporting, and may translate to professional reticence [35]. However, this is a relatively unexplored area and warrants further research.

- Wanting to remain “invisible”

URM students in particular may be less likely to report incidents. Our participants highlighted a desire to remain “invisible” and not be labelled as troublemakers. Ken, for instance, stated that he would never report anything unless full anonymity was guaranteed:

…I won’t ever fill out anything that’s not anonymous…[because] you just don’t want uni to see your face and mark you as a troublemaker.

This same sentiment was echoed by participants in Colenbrander, Causer and Haire [35] who cited being labelled as troublemakers as a barrier to reporting. This concern is not unfounded, as the most common form of institutional retaliation experienced by those who had reported an incident, especially cisgender men, was to be labelled a “troublemaker” [37]. This effectively dismisses the concerns of the reporter and shifts blame to them.

- Being a bystander

A final barrier to reporting occurs when students witness discriminatory incidences as bystanders. Many of our participants felt that they were unable to intervene due to the uncertainty of the impact it could have on their learning.

Because…she (the consultant) is teaching you, she’s senior to you…at that point, I don’t feel like I could have said ‘I feel like what you’re saying is wrong’ because I don’t know how that will go. So it’s just one of those situations where you have to either say nothing or uncomfortably laugh. — Sarina.

It’s really hard to challenge people’s opinions like, especially when they’re like a senior person. — Inaya.

The extent of intervention depends on how much bystanders identify with the organisation and their levels of safety within the organisation [38]. Participants previously reported a lack of belonging and faith in their respective institutions, explaining the hesitancy to intervene. Students also cited the hierarchy within medicine, as they felt unable to intervene when the perpetrator was a senior member of staff.

### How Students Compensated

#### ‘Strategic Flirting’

Students reported a myriad of ways to compensate with discriminatory behaviours and practices. For instance, many female participants mentioned using, and witnessed other women use, their femininity to accomplish certain tasks.

Emma elaborated on this and stated that “…you might as well use a bit of your charm to kind of…you know…be invited to a surgery…[or] be invited to learn three times more than anyone else…[or] be told you can go home when you wanna go.” She further described how a male senior even recognised and took advantage of this when needing to get a radiology scan protocolled. He asked Emma to go to a specific radiologist because “he likes girls and it will get done quickly.”

However, Shreya mentioned that she personally did not feel comfortable engaging in these behaviours, but she still felt under pressure to do so: “Every time I’m going to theatres I just always have this anxiety like I’m gonna have to like, really pick up my voice or be extra smiley just to get someone’s attention or to even be allowed in the room or, you know, be included…”.

There is a noticeable absence of research which explores ‘strategic flirting’, particularly in medical education literature. However, Henningsen’s [39] concept of “instrumental motivation” is seen in the above testimonies. Although outdated, this suggests the value of instrumentally motivated flirting. However, if this is as common as it was reported in our study, this may negatively impact students like Shreya who do not feel comfortable engaging in this behaviour. It also runs the risk of being misinterpreted as voiced by Emma, who stated that you had to “gauge” who you flirted with, recognising that caution must be taken when doing so as to not invite unwanted attention. More recent research is therefore required to better understand these behaviours and their effects in the current social climate.

#### Other Compensatory Behaviours and Participants’ Suggestions for Improvement

When questioned about compensatory behaviours, some participants described finding alternative teachers to approach. However, this took extra time which decreased productivity. Others described leaving the clinical environment and resorting to learning largely independently but noted that this was not as effective as they were not able to visualise anatomy or surgical procedures, or practice clinical skills. This demonstrates a gap between what these students were learning and what they could have learnt had they been treated differently during their surgical rotations, which suggests an area of further research.

**Fig. 1:**
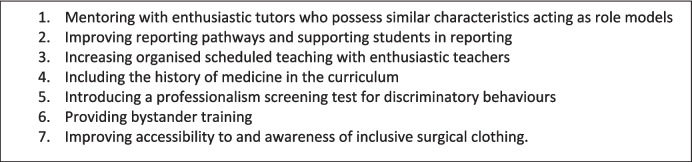
Suggestions for improvement

It is important to note that many participants stated that they were not able to adequately compensate for the impact that discriminatory behaviours had on their learning. They were, however, able to suggest numerous improvements to enhance the learning environment and reduce discriminatory behaviours. These suggestions can be subsumed under seven categories which are outlined below (Fig. 1), and expanded upon in Appendix D.

### Strengths and Limitations

Despite this study’s small sample size, which may have limited the generalisability, participants were of diverse backgrounds with intersections between different protected characteristics. This enabled us to capture a diverse range of experiences based on these characteristics. Furthermore, the modified-ethnography approach required frequent check-ins which not only provided researchers with a wide range of data which captured the more day-to-day experiences and perceptions, but also resulted in the formation of a more trusting relationship with the lead researcher. This trust then facilitated the emergence and in-depth discussion of more sensitive issues. The sample did not include participants with disability; gender reassignment; sexual orientation other than heterosexual; and there were no stated marriage or pregnancy-related issues. Further research should seek to include these protected characteristics.

It is important to recognise that participants may have been influenced by fears surrounding confidentiality and concerns regarding disapproval. These fears and concerns may have resulted in participants presenting only opinions that they believed were socially acceptable or less controversial. However, confidentiality was continuously reiterated throughout the research process to encourage participants to speak about their experiences.

## Conclusion

The clinical environment should be catering to the increasing diversity seen in its workforce. However, this study demonstrated discrepancies in the treatment students received based on their protected personal characteristics. It further highlighted how these experiences not only depend on categorical protective characteristics but also upon the way they these characteristics permeate and intersect with one another. Participants described the impact their experiences in the surgical environment had on their learning. Some students were able to identify ways in which they compensated for these discrepancies; however, many highlighted that compensation was not possible.

Although there is not yet strong evidence which supports specific interventions to reduce differential attainment, this study proposes ways in which this situation can be improved to optimise learning for students in the clinical environment based on discourse with participants and their experiences with reference to the relevant literature. Ideally this study would be replicated over a longer period with a larger sample size to improve generalisability and applicability of findings and to provide a deeper understanding of the complex issues that have been discussed. Improving the current situation should be of utmost importance, especially when considering the detrimental impact discriminatory behaviours can have on patient safety and the wellbeing of staff [40, 41].

## Appendix D.

Table 2

**Table 2:** Suggestions for improving the learning environment

Summary of suggestion	Explanation of suggestion
Mentoring with enthusiastic tutors who possess similar characteristics, acting as role models	Participants highlighted the value of being mentored by seniors who possess the same characteristics as them: “…if it was someone of a similar demographic…I just feel like they would understand the hindrances or like the like, obstacles that are sort of in your way while you’re trying to get into surgery. So I feel like they can relate to you more and they’ll be able to give you more advice. It’s more targeted and specific.” — Aisha “…when I’ve mentioned these [sexist] things to male colleagues, they don’t see it…So if I was to have a superior that was male, I wouldn’t feel necessarily as confident to have that conversation because you think am I just being hysterical? Is it just in my head? If you had a female supervisor you feel like she probably would have gone through worse and then she could probably relate to that.” — Shreya “So whenever you do see someone [with the same personal characteristics], I think it’s quite reassuring and you think, OK, well, she’s balancing it, you know. It’s nice to see.” — Sarina This is especially important when considering previous discussion surrounding how students believe seniors who possess the same characteristics as them tended to be more attentive to their learning needs. My findings demonstrate that seeing those with similar characteristics as them in areas where the students aspire to be is motivating and encouraging. However, students are not encountering these role models often enough due to the scarcity of certain demographics inhabiting leadership roles. Therefore, based on the experiences and suggestions of my participants, I propose that institutions or official surgical societies should be forming official mentoring programmes which connect students with an interest in surgery to surgeons. This should be prioritised on a personal characteristic basis, as our findings suggest that having similar personal characteristics as a student provides the mentor with invaluable relatability and insight into niche barriers and experiences. It also enables the student to view a surgical career as being attainable
Improve reporting pathways and supporting students	Multiple reasons have been provided for why students are not reporting discriminatory behaviours. These findings suggests that medical schools should be aiming to better educate students regarding reporting procedures and informing them of any updates to these, increasing transparency about outcomes, and reassuring them that their academic results will not be impacted. Universities should also be referring students to areas where they can seek pastoral support to help cope with any lasting impact of the incidents
Increase organised scheduled teaching with enthusiastic teachers	Many participants highlighted a lack of teaching in the clinical environment on the wards and often described being ignored. Therefore, I propose that institutions incorporate more scheduled bedside and theatre teaching with doctors who have expressed an enthusiasm to teach
Including the history of medicine into the curriculum	Ignorance was highlighted by participants when referring to those who participated in discriminatory behaviours. Alysha in particular referred to this in a racial context and spoke about an experience in which a doctor inferred that all black people do not seek medical attention early enough. Alysha spoke about how this may be due to how black people have been treated in the past by the medical field: “…when she left, I remember him saying to me about how it is so annoying seeing ‘these patients’ come so late when, like, she should have come earlier and stuff. And it was a black woman…He didn’t explicitly say it, but he was implying that it’s usually ‘them’ who don’t come early and in the back of my mind, I was also thinking there’s a historical reason why lots of black people are so hesitant to even interact with medicine in general. Like, if you think of the experiments that have been done and write up like the Tuskegee study, and there’s so much in it that people already kind of not too comfortable with medicine.” Therefore, I believe it would be beneficial to incorporate the history of medicine into the official curriculum, and to promote decolonisation of the curriculum to not only better understand the experiences of patients and colleagues and their behaviours but also to potentially reduce ignorance and in turn discriminatory incidents from occurring
Professionalism screening test for discriminatory behaviours	Participants voiced professionalism concerns about staff who participated in discriminatory behaviours, and spoke about witnessing their medical school colleagues also partake in such behaviours: “…it’s like when in med school when you come around students that just have very controversial views. It scares you to think that, OK, one day you’re going to be a doctor and have these views. So these [seniors] are the grown up versions of these students that you’ve come across and actually you don’t want to believe that people get that far and are able to treat people in a bad way.” — Sarina Alysha believed that this was because these behaviours were not explicitly “screened for” at any stage. This suggests value in utilisation or development of a screening tool that may identify such behaviours so that they may be appropriately dealt with to reduce professionalism concerns. This requires further research
Bystander training	The importance of the role of the active bystander has been highlighted in this study. Therefore, official bystander training should be provided to all students and staff to equip them to speak out and support others. Students and staff should also be educated on how to sensitively approach these issues, as teachers are often ill equipped to discuss characteristics such as ethnicity (Semlyen, et al., 2020), and white students have also expressed fear of causing offence (Roberts, Sanders, and Wass, 2008), which inhibits helpful discussion and impacts on intervention in the face of discriminatory events
Improve accessibility to and awareness of inclusive surgical clothing	The lack of awareness of, or accessibility to, inclusive surgical clothing such as the surgical hijab and surgical caps that cater to Afro-Caribbean hair was described in this study, as well the impacts of this. Therefore, I believe it pertinent to educate students and staff on the availability of these prior to commencement of surgical rotations. Such items of clothing should be incorporated into official hospital guidelines, and institutions should be advocating for this to occur, to decrease anxiety and provide clarity for their diverse cohorts. This may also reduce stigma associated with these items and make students feel catered for Institutions could also direct students to guides on how to maintain proper surgical attire whilst not compromising important aspects of their identity. Examples include guides produced by the British Islamic Medical Association (2022) and by Abdelwahab et al. (2021)
